# Casein and Peptides Derived from Casein as Antileukaemic Agents

**DOI:** 10.1155/2019/8150967

**Published:** 2019-09-08

**Authors:** Edgar Ledesma-Martínez, Itzen Aguíñiga-Sánchez, Benny Weiss-Steider, Ana Rocío Rivera-Martínez, Edelmiro Santiago-Osorio

**Affiliations:** Haematopoiesis and Leukaemia Laboratory, Research Unit on Cell Differentiation and Cancer, FES Zaragoza, National Autonomous University of Mexico, 09230 Mexico City, Mexico

## Abstract

Milk is a heterogeneous lacteal secretion mixture of numerous components that exhibit a wide variety of chemical and functional activities. Casein, the main protein in milk, is composed of *α*-, *β*-, and *κ*-caseins, each of which is important for nutritional value and for promoting the release of cytokines, also are linked to the regulation of haematopoiesis and immune response and inhibit the proliferation and induce the differentiation of leukaemia cells. It has been shown that the digestive process of caseins leads to the release of bioactive peptides that are involved in the regulation of blood pressure and the inhibition or activation of the immune response by serving as agonists or antagonists of opioid receptors, thus controlling the expression of genes that exert epigenetic control. Later, they bind to opioid receptor, block nuclear factor *κ*-beta, increase the redox potential, and reduce oxidative stress and the pro-inflammatory agents that favour an antioxidant and anti-inflammatory environment. Therefore, the bioactive peptides of casein could be compounds with antileukaemia potential. This review provides a summary of current knowledge about caseins and casein peptides on the immune system as well as their roles in the natural defence against the development of leukaemia and as relevant epigenetic regulators that can help eradicate leukaemia.

## 1. Introduction

Milk is a heterogeneous lacteal secretion mixture of numerous components (carbohydrates as oligosaccharides, lipids as long-chain polyunsaturated fatty acid, milk-specific microbiota, etc.) that exhibit a wide variety of chemical and functional activities. Milk is considered to be a functional food with direct and measurable influences on the health of the recipient [1], and it is now widely accepted that components of milk can influence and direct the physiological development of offspring.

In the traditional view, the major role of milk is to supply amino acids and nitrogen to young mammals, with use by adults being banned for most species; that is, humans are the only mammals known to consume the milk of another species, a unique behaviour that emerged during the Neolithic Revolution and that remains to this day. Thus, bovine milk has been an essential dietary staple for numerous human populations around the globe and an almost ubiquitous component of human nutrition [2, 3], regardless of the age of the consumer [4]. In this sense, among all mammals, bovine milk is the most studied; thus, we focus on it, especially the protein fraction of bovine milk that consists mainly of two major families of proteins, caseins (insoluble) and whey proteins (soluble), as well as other minor proteins and peptides, such as hormones.

We also discuss the available studies on breast milk and human caseins, since their data are relevant to the subject we address herein.

There are solid data indicating that caseins are linked to the immune system and to the generation of blood cells in mouse and rat models. Studies *in vitro* suggest that caseins, and the peptides resulting from the enzymatic hydrolysis of casein, have antitumour activity, which agrees with studies in humans that show that a lower frequency of breastfed infants develop leukaemia [5], and a similar effect has been described for older adults who consume milk of bovine origin [6], which suggests some factors that are transmitted through breast milk may prevent the development of this disease [7].

This narrative review provides a summary of current knowledge about caseins and casein peptides on the immune system and how they might have biomedical relevance in the defence against the development of leukaemia and as relevant epigenetic regulators that can help eradicate leukaemia.

All research articles for this paper were obtained by searching Google Scholar and PubMed (https://www.ncbi.nlm.nih.gov/pubmed/).

## 2. Intake of Milk and Cancer Risk

The intake of milk during childhood is fundamental since it is the only source of macro- and micronutrients [8] and since breastfeeding has a protective effect against infection in infants, and there are studies that suggest that breastfeeding confers protection against childhood cancer [9]. Mathur et al. examined the relationship between the duration of total breastfeeding and exclusive breastfeeding and childhood cancer (58 % of lymphoma cases were non-Hodgkin's lymphoma). Their results suggest that breastfeeding has a protective effect against childhood cancer. Furthermore, they indicate that exclusive breastfeeding provides more beneficial immunological effects than breastfeeding that is supplemented by alternative feeding [10].

Shu et al. tested the hypothesis that breastfeeding decreases the risk of childhood leukaemia in two case-control studies of childhood acute myeloid leukaemia (AML) with the M0, M1, and M2 morphologic subtypes, and for childhood early pre-B-cell lymphoblastic leukaemia (ALL). They show a reduction in risk among breastfed infants, particularly those breastfed for more than 6 months [5]. Few studies have explored the association between diet and adult AML. It has been shown that consumption of whole milk increases lung and ovarian cancer risk [11, 12], but the role of dairy products such as milk in the risk of cancer is inconclusive [13]. Thus, in a hospital-based case-control study of 111 cases and 439 controls, regular milk intake was a factor associated with a significant decrease in the risk of AML in females with the highest weekly intake of milk compared with those in the lowest intake category [13]. A multicentre case-control study was conducted in southeastern and northeastern China, and their findings suggest that diets rich in vegetables and an adequate amount of milk reduce the risk of adult leukaemia [6]. Additionally, milk intake has been related to a reduced risk of cancers of the distal colon and rectum [14]. These epidemiological data suggest that some component of milk has an antitumour effect but the composition of milk changes constantly throughout the lactation period and it has been shown that there are significant differences in milk composition between different species. Diet and the environment are important factors that influence the composition of milk. Some micronutrients may vary with nutritional status, and environmental toxins would differ according to the level of environmental exposure of chemicals specific to the region [10]. Further research is warranted to investigate the risk associated with milk intake.

## 3. Milk Composition

Milk contains specific proteins, fats designed to be easily digested, carbohydrates, minerals, vitamins, and other components [15]. Their composition reflects the nutritional requirements for the growth and development of each species. Thus, bovine milk is composed of approximately 3.2% protein, 4% lipid, 5% carbohydrates, and 0.7% mineral salts [16], whereas human milk consists of 1% protein, 4% lipid, 7% carbohydrates, and 1% mineral salts [17] (Table 1).

Milk protein has a high biological value, and milk is therefore a good source of essential amino acids; however, a wide array of milk proteins have biological activities that range from antimicrobial functions to the facilitation of nutrients absorption, and others act as growth factors, hormones, enzymes, antibodies, and immune stimulants [8].

Milk proteins can be broadly classified into 3 categories: caseins, whey proteins, and mucins, which are present in the milk fat globule membrane. In milk, caseins interact with calcium phosphate, forming large stable colloidal particles termed micelles. These micelles make it possible to maintain a supersaturated calcium phosphate concentration in milk, providing the newborn with sufficient calcium phosphate for the mineralization of calcifying tissues [21].

Milk proteins also facilitate the uptake of several important nutrients such as trace elements and vitamins and contain a group of proteins that provide a protective function, indicating their importance as multifunctional substances [22].

Bovine whey protein comprises immunoglobulins, *α*-lactalbumin, *β*-lactoglobulin, serum albumin, immunoglobulin, lactoferrin, proteose peptone fractions, and transferrin. Lower amounts of other minor proteins and peptides also exist with, for example, hormonal or other physiological activities [23]. In human milk, the whey proteins found in significant quantities are *α*-lactalbumin, lactoferrin, IgA, osteopontin, and lysozyme [18].

Bovine caseins, the most thoroughly studied, comprise *α*s1-, *α*s2-, *β*-, and *κ*-caseins. They are synthesized in the mammary gland under multihormonal control, and in the bovine genome, they are associated within a 200 kb region on chromosome 6, in the following order: *α*s1-, *β*-, *α*s2-, and *κ*-casein [24].

*β*-Casein has 209 amino acids. The presence of proline or histidine at the 67th position of *β*-casein allows the distinction between two types of milk, A1 and A2, and there are no other differences between these caseins. A1 *β*-casein is a major variant of *β*-casein in the milk of the common dairy cows of north European origin: Friesian, Ayrshire, British Shorthorn, and Holstein. A2 *β*-casein is predominantly found in the milk of Channel Island cows, Guernsey and Jersey, in Southern French breeds, Charolais and Limousin [25], and in the Zebu original cattle of African origin. The presence of proline or histidine at the 67th position of *β*-casein is associated with the major effects from bioactive peptide release by different gastrointestinal enzymes [26]; thus, a bioactive seven-amino-acid peptide, *β*-casomorphin-7 (BCM7) can be more easily released by digestion in the small intestine of A1 *β*-casein with pepsin, leucine aminopeptidase, and elastase, but the alternative proline at position 67 prevents protein cleavage at this site [27]. There is a hypothesis that A1 (but not A2) *β*-casein may increase the risk of developing type I diabetes (DM-I) in genetically susceptible children [28], and it was suggested that A1 *β*-casein may also be a risk factor for coronary heart disease (CHD) [29].

*α*s2-Casein constitutes as much as 10 % of the casein fraction in bovine milk; it consists of 2 major and several minor components that exhibit various levels of post-translational phosphorylation [30], as well as minor degrees of intermolecular disulfide bonding [31].

*α*s2-Casein is the most calcium-sensitive member of the casein family, possibly because of its high ester phosphate content, which is derived from 10 to 13 phosphate groups on each peptide chain [32].

## 4. Caseins as Regulators of Haematopoiesis and the Immune System

Historically, sodium caseinate (SC), a bovine casein salt soluble in water with 65% proteins [33], provided the first evidence that milk proteins are linked to the biology of the immune system. SC used as a pro-inflammatory molecule induces chemotaxis of granulocytes and macrophages in the peritoneal cavity of mice [34, 35] and induces the accumulation of myeloid progenitor cells in mouse bone marrow [36]. Over time, it has been shown that SC accelerates the transition of band cells from bone marrow to polymorphonuclear cells, thus inducing macrophage colony-stimulating factor (M-CSF) [37]. Bone marrow progenitor cells of mice cultured with interleukin 3 (rmIL-3) as a growth factor in the presence of SC show increased cell numbers that exceed 50% [38]. Consequently, the administration of SC every 48 h for 6 days in BALB/c mice has been shown to increase myeloid cell proliferation and the number of total and mononuclear cells from bone marrow, events that are considered indices of medullary haematopoiesis activation [36]. SC also induces the proliferation of granulocytic lineage cells and increases the levels of both granulocyte colony-stimulating factor (G-SCF) and granulocyte macrophage colony-stimulating factor (GM-CSF) cytokines in serum and of G-CSF in bone marrow plasma, and the granulocytes generated have enhanced phagocytic activity [39]. This enhanced granulopoiesis and the subsequent reinforcement of innate immune system activation could explain why mice injected with lethal doses of bacteria survive after administration of casein [40].

Since SC consists of *α*-, *β*-, and *κ*-casein molecules, it seems logical to conclude that caseins would have biological effects similar to those of sodium salt. Most milk proteins are susceptible to the degradative effects of gastric processing, and extensive hydrolysis takes place upon exposure to enzymes in the gut; therefore, there are a few reports that show the biological effect of bovine and human caseins as complete molecules *in vitro*, without previous enzymatic degradation (Table 2).

Evidence has shown that caseins *in vivo*, through the production of cytokines, could be involved in the development of the mucosal immune system in neonatal mice [49], in erythropoiesis of mice [50], and in the restoration of haematopoiesis in rat models of myelosuppression [51].

## 5. Systemic Effect of the Peptides Derived from Milk Proteins

It has become increasingly evident that a consideration of the milk protein value must take into account the relationship between the protein structure and the amount and composition of the peptides derived from the proteins casein and lactalbumin during digestion in the gastrointestinal tract [52]. In addition, the relevant physiological activities of the infant during the breastfeeding and of children and adults consuming bovine milk should be considered.

Biologically active peptides derived from milk proteins are defined as fragments of 3–20 amino acid residues that have a positive impact on the physiological functions of the body. In general, these peptides are inactive within the sequence of the parent protein; thus, functional properties are revealed only after degradation of the native protein structure during gastrointestinal digestion or food processing. Once they become a bioactive, peptides may act as regulatory compounds with hormone-like activity [22], which ultimately affects the health of the living organism [53].

This gastrointestinal degradation may be a consequence of enzymatic hydrolysis, fermentation of milk by the starter cultures of proteolytic bacteria, and other processes used in dairy production [54]. In most cases, caseins are enzymatically degraded in the gut by endogenous enzymes, secreted by the digestive system, by enzymes of exogenous origin derived from actively metabolizing gut microflora [52] or, alternatively, by enzymatic degradation of granulocytes and macrophages [55, 56]. In any case, many of the peptides released by enzymatic hydrolysis have specific biological functions on their basis of their ability to bind to (and affect) the cellular function [57, 58].

Bioactive milk peptides were described for the first time after studies showed that the ingestion of casein-derived phosphorylated peptides led to enhanced vitamin D–independent calcification in rachitic infants. Since this discovery, several immunomodulatory peptides have been found in bovine and human milk [23, 59, 60]. Among the most studied peptides are *α*-lactalbumin derivatives, as well as *α*-, *β*-, and *κ*-caseins (Table 3). The following peptides have been studied: (1) Casein phosphopeptides, generated by the degradation of *α*- and *β*-caseins, are involved in promoting the absorption of calcium in the intestine to simulate the calcification of bones [76]. (2) Peptide inhibitors of angiotensin-1 converting enzyme, derived from *α*-lactalbumin, and *α*- or *β*-casein are crucial for regulating blood pressure [77]. (3) Opioid agonists derived from *α*- or *β*-casein and *α*-lactalbumin play an important roles in sleep patterns and are necessary for the development and gastrointestinal function on infants [78]. (4) Antioxidant peptides of *β*- or k-casein eliminate reactive oxygen species by reducing oxidative stress in newborns [79–81]. (5) Immunostimulatory peptides of *β*-casein and *α*-lactalbumin stimulate the phagocytic activity of macrophages [64, 82]. (6) The *α*-lactalbumin peptides exhibit bactericidal activity, since they have a high affinity for the iron in pathogens, which they use to exert a strong bacteriostatic effect [68, 78].

All these studies suggest to us that these systemic repercussions after milk intake in human beings, maternally sourced during the first months of life and of bovine origin in childhood and adulthood, could be of medical and clinical interest, and special attention might be directed to studies on opioid peptides.

## 6. Opioid Peptides from *β*-Casein, *α*-Casein, and *κ*-Casein

Opioid peptides are defined as peptides such as enkephalins that have both affinities for opiate receptors and opiate-like effects that are inhibited by specific antagonists of opiate receptors such as naloxone. The typical opioid peptides all originate from three precursor proteins: proopiomelanocortin (endorphins), proenkephalin (enkephalin), and prodynorphin (dynorphins) [83]. All of these typical endogenous opioid peptides have the same N-terminal sequence: Tyr-Gly-Gly-Phe [22]. On the other hand, food protein–derived opioid peptides are classified as exogenous opioids: while they possess a Tyr residue within their sequence, usually at the N-terminus or in the N-terminal region (except for *α*s1-CN-exorphin, casoxin 6, and lactoferroxin B and C), they differ from endogenous opioid peptides, which often feature Tyr-Gly-Gly-Phe as the N-terminal sequence [84], potentially with another aromatic residue, Phe or Tyr, at the 3rd or 4th position [85]. It is thought that, as in endorphins, this domain is important for the binding of peptides to the opioid *µ*-receptor (MOR) in the central nervous system, gastrointestinal tract, and some immune cells [86, 87]. In addition to its structural similarity, the activity of peptides is abrogated by naloxone, and therefore, it is accepted that these milk peptides affect the opioid receptor pathway [58, 88].

In most cases, these exogenous peptides were isolated and subsequently identified from enzymatic digests of their parent protein molecules. All the major milk proteins contain opioid ligands, which have been specifically termed exorphins and casoxin D when derived from *α*-casein.

Other milk opioid agonist peptides are *α*-casein-derived exorphins corresponding to bovine *α*s1-casein f90–95 (Arg-Tyr-Leu-Gly-Tyr-Leu) and f90 ± 96 (Arg-Tyr-Leu-Gly-Tyr-Leu-Glu), both of which have opioid-like properties that are inhibited with naloxone [1].

*β*-casomorphins (BCMs) are 4 to 11 amino-acid peptides encrypted in an inactive form and are released during digestion both *in vivo* and *in vitro*. Among them, the most active are BCM7 and BCM5, which represent fragments f60–66 and f60–64 of *β*-casein, respectively [89]. Both of these BCMs cross the intestinal barrier and reach the cerebrospinal fluid in normal individuals [90]. The physiological implications of this phenomenon have not yet been clarified, but it has been suggested that there is a relationship between BCMs and autism. *β*-casomorphin induces Fos-type immunoreactivity in brain regions relevant to autism, and elevated levels of BCM7 have been observed in patients with this condition in whom it exerts a relaxing effect [90]. Similarly, BCM7 and opioid receptors could be related to schizophrenia in people with few opioid receptors [91]. Thus, despite high levels of BCM7 in these patients [90], BCM7 cannot exert the relaxing effect it does in autistic patients.

BCMs were originally isolated from human and bovine *β*-casein following trypsin hydrolysis *in vitro* [92]. Pepsin and LAP are responsible for the release of the Tyr residue at the N-terminus of all types of pro-BCMs: pepsin cleaves the Leu58-Val59 peptide bond and LAP removes valine from the amino terminus. It should be noted that these peptides show strong opioid activities after the valine residue is removed [93].

BCM inhibits the proliferation of human lamina propria–derived lymphocytes *in vitro*. This antiproliferative effect is reversed by the addition of the opiate receptor antagonist naloxone to the culture [92]. However, BCM also enhances the resistance of mice to *Klebsiella pneumoniae*, likely by stimulating peritoneal macrophages. Additionally, the administration of an opioid antagonist in mice *in vivo* results in the suppression of this stimulatory effect, suggesting an active opioid receptor binding site for the biologically active peptide [42].

BCM7 f60 ± 66 and BCM10 f193 ± 102 (Tyr-Pro-Phe-Pro-Gly-Pro-Ile and Tyr-Gln-Gln-Pro-Val-Leu-Gly-Pro-Val-Arg, respectively) can exhibit bipolar modulatory effects on human peripheral blood lymphocyte proliferation. In *in vitro* cultures with mitogen-stimulated T lymphocytes, both peptides at low concentrations have been shown to suppress proliferation but enhance proliferation when administered at high concentrations [71].

## 7. Caseins and Peptides Derived from Caseins in the Regulation of Cancer

A wide variety of bioactivities for milk protein components has been reported, with one component having more than one type of biological activity, but here, we present only examples in which caseins and casein peptides have effects on different cancer cell lines or animal models. Then, we focus on the antileukaemic activities of these peptides. *α*-, *β*-, and *κ*-casein proteins all inhibit the migration *in vitro* of murine mammary tumour cells of the Met-1 cell line, the human breast cancer cell line MCF10A-H-Ras (G12V), and MDA-MB-231 cells, with *α*-casein being the most effective [94].

Casein hydrolysates generated using different commercially available food-grade enzyme preparations from mammalian, bacterial, and plant sources have an inhibitory effect on the viability and growth of both human Jurkat leukaemia T-cells and human epithelial colorectal adenocarcinoma Caco-2 cells lines, but SC had no significant effect on the viability and growth of Caco-2 cells [95].

Peptides derived from *α*s1-casein and *β*-casein digested by lactic acid bacteria inhibit the enzymatic activities of purified recombinant matrix metalloprotease (MMP)-2, MMP-7, and MMP-9 in human HT-29 and SW480 colon carcinoma cells [96].

Lactaptin, the proteolytic fragment (f57 ± 134) of human *κ*-casein, induces apoptosis of MCF-7 adenocarcinoma cells [97]. Additionally, RL2, a recombinant analogue of lactaptin, induces apoptosis in MDA-MB-231 cells from an epithelial human breast cancer cell line and MCF-7 cells, and both downregulates Bcl-2 expression and induces p53-independent cell death [98]. On the another hand, it reduces the viability of A549 lung carcinoma cells and Hep-2 larynx epidermal carcinoma cells but is not accompanied by apoptosis, and in an interesting finding, nonmalignant human mesenchymal stem cells (MSC) are completely resistant to the action of RL2 [99].

In addition, 90-95 and 90-96 *α*-casomorphin, BCM7, BCM5, and the morphiceptin, the amide of *β*-Casomorphin-4, have an antiproliferative action on T47D cells, blocking cells in the G0/G1 phase [58].

Furthermore, 90–95 and 90–96 *α*-casomorphin, BCM5, and *α*s1-casomorphin amide inhibit the proliferation of human prostate DU145 and PC3 cells [100].

Moreover, f63-68 from *β*-casein inhibits the proliferation of SKOV3 human ovarian cancer cells partially by promoting apoptosis through suppression of the BCL2 pathway [101]. *β*-casein peptide f41-45 induces cytotoxicity in B16F10 melanoma cells [102].

Among the first findings of antitumour activity of casein *in vivo*, rats fed a diet rich in casein showed a marked decrease in colon carcinogenesis that had been induced by azoxymethane compared with the carcinogenesis in rats fed a low casein diet [103].

RL2, a recombinant analogue of lactaptin, significantly suppressed the growth of solid tumours in mouse xenografts bearing MDA-MB-231 breast cancer cells [98].

## 8. Caseins and Peptides Derived from Caseins Have Antileukaemic Properties

The first evidence of the antileukaemic activity of protein milks was shown *in vitro* by SC inhibiting the proliferation of leukaemia in mouse cells, such as those from the WEHI-3, J774, and P388 cell lines, even inducing apoptosis in one of them: the WEHI-3 myelomonocytic leukaemia cell line. However, in mononuclear normal cells from BALB/c mice (MNCs) bone marrow, SC induces a marked proliferation stimulus [38]. The evidence showed that normal tissues could be less sensitive to the biological effects of new molecules with potential antileukaemic properties [104, 105]; these data are significant since the usefulness of a potential anticancer compound depends not only on its ability to induce cytotoxicity in malignant cells but also on its relative lack of ability to induce toxicity in normal tissues and, in the case of SC, its ability to suppress the proliferation and induce the death of leukaemia cells. However, in addition to exerting no cytotoxicity towards nonleukaemia MNCs, SC induces their proliferation, which is a rare property among most drugs tested for use in the treatment of acute myeloid leukaemia. Then, it became clear that not only caseins but also casein peptides had an inhibitory effect on the proliferation of leukaemia cells when the casein hydrolysate inhibited the proliferation of the J774 and P388 leukaemia macrophage-like cell lines, although only in the latter was cytotoxicity confirmed [106]. Other evidence suggest that *κ*-casein f25-34 and f35-41 inhibit the proliferation of 32D normal cells and WEHI-3 myelomonocytic leukaemia cells and induce the differentiation of cells in the monocyte-macrophage and granulocyte-neutrophil lineages. *κ*-casein f35-41 reduces the proliferation of cells in both cell lines and induces 32D differentiation towards the monocyte-macrophage lineage, and WEHI-3 cell differentiation towards the granulocyte neutrophil lineage, whereas *κ*-casein f58-61 has no effect on the proliferation of any of the cells but induces their differentiation towards becoming granulocytes in both cell lines. This reduced proliferation is not due to a possible cytotoxic effect of the molecules [107].

*β*-casomorphin decreases the proliferation of 32D mouse cells by as much as 50% [108], and suppresses the proliferation of cells in the WEHI-3 myelomonocytic leukaemia cell line [109].

It was later shown that SC injected i.p. into mice inoculated lethally with WEHI-3 myelomonocytic leukaemia cells reduced the tumour burden and suppressed hepatomegaly, which collectively increased the survival of the leukaemic mice to a significant extent [38]. Similarly, in mice inoculated with cells from the J774 leukaemia macrophage-like cell line, a model of macrophage-like tumour M5 AML, SC significantly reduced splenomegaly, hepatomegaly, and the presence of solid tumours [110]. In both cases, the mechanisms of this antileukaemic action *in vivo* are unknown, but it has been observed that i.p. administration of SC in healthy mice induces the production of cytokines both in plasma and bone marrow [39]; therefore, the antitumour effects of SC might be the result of induction of profound inflammatory cell migration into the peritoneal cavity [34], either via the bioactive components of SC [111] or the secretion of growth factors, cell differentiation, or the effect of systemic inflammation [40]. Additionally, SC can activate mechanisms other that those associated with a simple inflammatory process because, although other agents, such as zymosan or thioglycolate, increase the levels of pro-inflammatory cytokines (IL-1*β*, TNF-*α*, MIP-2, and MCP-1/CCL2) [112, 113], they have no inhibitory effect on the proliferation of haematopoietic cells [114].

The available evidence for caseins, both in their complete form and in fragments resulting from their enzymatic degradation, reveal an enhancement of different aspects of the immune system, but their potential as antitumour agents has been scarcely explored. The use of caseins or their peptides to enhance the immune system to fight cancer is a rational strategy, as the immune system constantly works to keep us free of tumours. However, it is, of course, not always successful, with an estimated 19,520 new cases of AML diagnosed in the United States in 2018, accounting for approximately one-third of all new leukaemia cases [115]. Nevertheless, enhancing the immune system to eradicate cancer remains a valid and widely explored strategy against cancer. There are elements that suggest that caseins or casein peptides could eradicate leukaemia by functioning as enhancers of the immune system and inducing cell death of malignant cells.

As we noted above, the mechanisms of the antileukaemic action of SC *in vivo* are unknown, but all these data on casomorphins, added to the fact that both granulocytes and macrophages are capable of hydrolysing caseins to release biologically active peptides [55, 56], suggest to us that these opioid peptides may be responsible for the antileukaemic effects observed for SC or caseins [116].

## 9. Mechanism of Action for Caseins and Derivate Peptides in Haematopoietic and Leukaemia Cells

It has been shown that in haematopoietic cells such as polymorphonuclear cells and monocytes there are specific receptors for caseins [117, 118], although little attention has been paid to this topic and it has been looked at whether another type of receptor in haematopoietic cells could be involuted in the biological effects of caseins has been explored.

**Figure 1:**
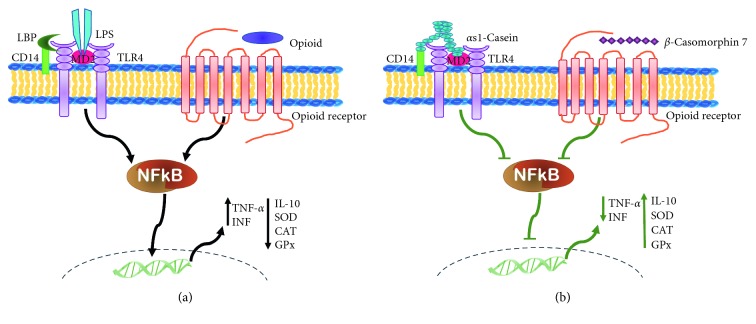
Mechanism of antineoplastic activity induced by casein or BCM7. (a) OPRs or TLR4 ultimately activates the nuclear factor *κ*-beta (NF*κ*B) and the main proinflammatory promoter that prevails in the tumour microenvironment and increases in proinflammatory cytokines such as TNF-*α*, IL-1*β*, and IFN-γ but reduces anti-inflammatory molecules such as IL-10, SOD, CAT, and GPx. (b) BCM7 activates the MOR or *α*s1-casein, activates TLR4, and reduces the activation of NF*κ*B, reduces the levels of TNF-*α* and IFN-γ, and increases IL-10, SOD, CAD, and GPx, contributing to a weakened leukaemogenic environment.

Haematopoietic stem/progenitor cells (HSPCS) and their differentiated progeny express toll-like receptors (TLRs), which ensure an effective immune response in response to acute damage or infection. They are also responsible for promoting the recognition and elimination of tumour cells. Consequently, the recognition of TLR4 on antigen-presenting cells enhances antigen-specific antitumour immunity [119, 120], and an immunotherapeutic regimen capable of eliminating large, established mouse tumours has been developed using HMGN1, a DC-activating TLR4 agonist that is capable of inducing antitumour immunity [121]. It has recently been shown that *α*-casein binds to TLRs [47, 122]; thus, casein could exert immunomodulatory effects on leukocytes and even participate in the genesis of blood cells via TLRs (Figure 1), which could explain the antineoplastic effect of *α*-casein in WEHI-3 leukaemia cells [116].

However, overexpression or aberrant translation of TLR signalling is also associated with inefficient or malignant haematopoiesis, as in the case of leukaemia. Thus, overexpression of TLR-4 and TLR-2 has been observed in acute myeloid leukaemia and is more pronounced in acute promyelocytic leukaemia (Table 4), but it is reduced in the cells of patients treated with chemotherapy, suggesting the involvement of cellular signals that promote the development and prevalence of leukaemia [128, 131]. The activation of TLRs induces the production of interleukin 8 (IL-8), which attracts suppressor cells derived from the myelocytes (MDSCs) responsible for propitiating a tumorigenic microenvironment [132]. Additionally, leukaemia cells have been shown to stimulate bone marrow stromal cells of one-self to produce IL-8, a cytokine that supports the development of leukaemia cells [132]. During signal transduction, TLR activates nuclear factor *κ*-beta (NF*κ*B) [133], the main pro-inflammatory promoter prevailing in the tumour microenvironment, leading to increases in pro-inflammatory cytokines such as TNF-*α*, IL-1*β*, and IFN-γ but reductions in anti-inflammatory molecules such as interleukin 10 (IL-10), SOD, CAT, and GPx (Figure 1(a)). Thus, the role of TLRs in the genesis and/or elimination of leukaemia is controversial; therefore, it is pertinent to suggest that more studies are needed to clarify the circumstances under which the TLRs are associated with the development of cancer and under what conditions these same receptors can serve as a therapeutic alternative against the development of leukaemia.

We know that SC administration in the peritoneal cavity promotes the survival of leukaemic mice [38, 110]. The possible mechanism of this antileukaemic effect may be due to the activation of TLRs by *α*-casein to exerting an antitumour activity (Figure 1(b)). Alternatively, these resident cells of the peritoneal cavity of mice could induce casein fragmentation to release casomorphins with opioid receptor activities, as indicated below.

It has been observed that the absence of MOR in mice enhances the genesis of haematopoietic progenitor cells [134], revealing a possible negative regulatory role of haematopoiesis for this type of receptor. In contrast, overexpression of this type of receptor has been observed in leukaemia cells (Table 4), and the use of opioid agonists has even been proposed for the treatment of different types of tumours, including those of leukaemia [135]; in this sense, methadone, a specific ligand of MOR, has been proposed for the treatment of cancer [135, 136] because it induces apoptosis and increases the sensitivity of leukaemia cells to the effect of doxorubicin in a mechanism that involves the reduction of cAMP, a promoter of cell proliferation [124].

In the framework of the development of leukaemia, in addition to high levels of reactive oxygen species (ROS), the expression and activation of antioxidant enzymes such as superoxide dismutase (SOD), catalase (CAT), and glutathione peroxides (GPx) are disturbed, in particular, both SOD and CAT activity are reduced in lymphocytes from ALL and CLL patients [137, 138]. It has been shown that BCM7, using epigenetic control, elevates the levels of glutathione S transferase (GST), a detoxifying enzyme of cancer-promoting agents [139], which is expressed at low levels in patients with leukaemia [140]. In fact, the GST gene is hypermethylated in the lung, breast, and liver cancers; leukaemia; and lymphomas [141].

After 15 days of oral administration of BCM7 to diabetic mice, the pancreatic malondialdehyde level was markedly reduced, with an increase in CAT activity and a reduction in NF*κ*B and iNOS gene expression. Thus, BCM7 causes a pronounced decrease in oxidative stress and inhibits the NF*κ*B-iNOS-NO signalling pathway [142]. Additionally, it has been shown that BCM7, when binding to MOR, increases the GSH/GSSG ratio and decreases the level of enzymes involved in SAM/SAH methylation, resulting in a reduced methylation of the CpG region [139], revealing its role as an epigenetic modulator of relevant genes in redox control.

All these elements suggest that BCM7 could decelerate leukaemogenesis via MOR, or, as an alternative, CN and *α*s1-caseins via TLR4. In any case, as a consequence of the activation of either receptor or both of them, the signalling of NF*κ*B, the main pro-inflammatory promoter that prevails in tumour microenvironments, could be blocked, thus reducing the levels of pro-inflammatory cytokines but increasing anti-inflammatory molecules, which could reduce the leukaemogenic environment (Figure 1(b)).

## 10. Future Perspectives

It is undeniable that leukaemia cells overexpress TLRs and opioid receptors that bind caseins and casomorphins, respectively. In both cases, the interaction leads to a reduction in the pro-inflammatory microenvironment prevalent in the development of tumours, so it would be interesting to evaluate whether by effectively reducing the oxidative stress, the production of anti-inflammatory cytokines is favoured over the production of pro-inflammatory cytokines; such information would support the potential antioncogenic use of caseins and casomorphins.

Caseins and some casomorphins inhibit proliferation and induce the differentiation of leukaemic but not normal cells, and caseins promote proliferation and differentiation of cells and even prolong the survival of leukaemic mice. It would be very interesting to determine whether the cause of these biological effects depends on the presence of TLRs and/or MOR.

Given the relevance of the physiological effect of the peptides derived from casein, it is reasonable to consider that they can have a relevant role as micronutrients and that their absence can cause the development of not only leukaemia but also of another types of cancers.

It should not be overlooked that *α*S1-casein is expressed in cells distinct from the mammary gland, mainly in patients with autoimmune diseases, which makes it necessary to analyse with caution the role of this compound as an antineoplastic agent.

## 11. Conclusions

There is evidence that caseins, both in their complete form and in fragments produced by their enzymatic degradation, enhance different aspects of the immune system, such as the proliferation of lymphocytes and generation of antibodies. They can also regulate normal haematopoiesis *in vitro* and *in vivo* via the secretion of cytokines, thereby inducing differentiation and enhancing proliferation. In leukaemia cells, however, they induce apoptosis and negatively regulate proliferation. This phenomenon highlights the potential of milk proteins as antitumour agents, but further research is needed to fully understand the mechanisms underlying the effects of the bioactive peptides of milk. Thus far, we have been shown that the TLR and OPR are involved in the transduction of signals from casein peptides in leukaemia and normal haematopoietic cells. Although humans consume milk over a much longer period than other mammals, we do not yet understand the complete scope of the administration of casein or its peptides as an antileukaemia therapeutic regimen. However, the ultimate proof that a milk-derived product will or will not benefit human health will only be obtained in clinical trials.

## Figures and Tables

**Table 1:** Differences in the composition of human and bovine milk.

	Human	Bovine	Ref
Protein	Predominantly whey and *β*- and *κ*-caseins with lower concentrations of *α*-casein	The major protein fractions consist of *α*-, *β*-, and *κ*-casein	[17, 18]

Lipid	Cholesterol, palmitic and oleic acids, phospholipids, arachidonic acid, eicosapentaenoic acid, docosahexaenoic acid, and long-chain polyunsaturated fatty acids (APGI-LC)	Triacylglycerols 98.3%, diacylglycerols 0.3%, monoacylglycerols 0.03%, free fatty acids 0.1%, phospholipids 0.8%, and sterols 0.3%	[19, 20]

Carbohydrate	Mainly lactose (6–8 g/100 ml) but at least 30 oligosaccharides, all of which contain terminal Gal-(*β* 1,4)-Glc and range from 3–14 saccharide units per molecule	In addition to lactose (4–6 g/100 ml), oligosaccharides, glycoproteins, and glycolipids	[15, 19]

Minerals	Calcium 25–35 mg/100 ml Phosphorus 13–16 mg/100 ml Sodium 15 mg/100 ml Potassium 156 mg/100 ml	Calcium 120 mg/100 ml Phosphorus 94 mg/100 ml Sodium 43 mg/100 ml Potassium 58 mg/100 ml	[20]

Vitamins	Retinol 58 *μ*g/100 ml Vitamin E 0.34 mg/100 ml Biotin 0.7 *μ*g/100 ml Riboflavin 0.03 mg/100 ml Vitamin B6 0.01 mg/100 ml Vitamin B12 trace	Retinol 19 *μ*g/100 ml Vitamin E 0.04 mg/100 ml Biotin 3 *μ*g/100 ml Riboflavin 0.24 mg/100 ml Vitamin B6 0.06 mg/100 ml Vitamin B12 0.9 mg/100 ml	[15, 20]

**Table 2:** Effect of caseins in haematopoietic cells *in vitro*.

Casein	Biological functions	Ref
Bovine *α*-, *β*-, and *κ*-caseins	Inhibit the proliferation of the 32D myeloid mice cell line and induce the expression of cfms and FcgRIIB1 and FcgRIIB22 receptors	[41]
Bovine *α*-, *β*-, and *κ*-caseins	Inhibit the proliferation of WEHI-3 leukaemic cells but induces cell differentiation, the expression of GM-CSF and its receptor GM-CSFR, as well as the isoforms FcgRIIB1 and FcgRIIB22	[42]
Human *α*S1-casein	Activates the secretion of pro-inflammatory cytokines such as GM-CSF, IL-1*β*, and IL-6 in human monocytes via the MAPK-p38 signalling pathway	[43, 44]
Human *α*S1-casein	Enhances the mitogen-stimulated proliferation of murine splenic T lymphocytes	[45]
Human *α*S1-casein	Pro-inflammatory properties throughout the TLR4 pathway	[46]
Human *α*S1-casein	May constitute an autogenous stimulus to uphold chronic TLR4 pathway inflammation	[47]
Bovine *β*-casein	Enhances mitogen-induced proliferation of bovine T and B lymphocytes in a dose-dependent manner	[48]
Bovine *κ*-casein CGP	Suppresses murine and rabbit lymphocyte proliferation induced by mitogens	[45]

cfms, M-CSF receptor; IL-1*β*, interleukin 1*β*; IL-6, interleukin 6; CGP, caseinoglycopeptide; MAPK-p38, mitogen-activated protein kinase p38.

**Table 3:** Immune activities of peptides and protein hydrolysates from caseins.

Casein	Derived peptide	Biological functions	Ref
*α*s1-Casein	Trypsin-derived f194-199 C-terminal	Promotes antibody formation and accelerated phagocytosis *in vitro* Provides protection against lethal bacterial infections *in vivo* Reduces *Klebsiella pneumoniae* infection in mice *in vivo*	[61, 62]
*α*s1-Casein	Chymosin-derived f1 ± 23 N-terminal	Protects mice against infection by *Staphylococcus aureus* prior to infection Stimulate a phagocytic response in mice infected with *Candida albicans* when injected intravenously Protection in cows and sheep against mastitis has also been observed following injection of the peptide into the udder	[63]
	Caseins digested by non-pretreated trypsin	Stimulate phagocytosis by murine peritoneal macrophages *in vitro* and consequently to exert a protective effect against *K. pneumoniae* challenge in mice after intravenous treatment	[64]
*α*s1-Casein	Pepsin/trypsin-derived peptides	Inhibit the proliferative responses of murine splenic lymphocytes and rabbit Peyer's patch cells *in vitro* Suppress mitogen-induced proliferation of human peripheral blood mononuclear cells *in vitro*	[65]
*α*-Casein	dPHLr	Decreases the production of IL-2 in activated T lymphocytes *in vitro*	[66]
*α*s1-Caseins	HLGG	Suppresses the proliferation of lymphocytes	[65]
*κ*-Casein∗	Synthetic peptide Tyr-Gly	Enhances the proliferation of human peripheral blood lymphocytes *in vitro*	[67]
*κ*-Casein∗	Chymosin-derived f106 ± 169 CGP	Inhibits LPS- and PHA-induced proliferation of murine splenic lymphocytes *in vitro*, and it also suppresses antibody production in murine spleen cell cultures *in vitro*	[48]
*κ*-Casein	Pepsin/trypsin-derived peptides	Enhances mitogen-induced proliferation of human lymphocytes *in vitro*	[68]
*κ*-Casein	Trypsin-derived f17 ± 21	Promotes antibody formation and accelerated phagocytic activity of murine and human macrophages *in vitro*	[69, 70]
*κ*-Casein	Synthetic peptide f383-389 (Tyr-Gly)	Immunomodulating peptide can pass across the intestine in quantitatively significant amounts to reach local lymphocytes Enhances cellular proliferation of human peripheral blood lymphocytes activated with concanavalin A *in vivo*	[71, 72]
*β*-Casein	FLAb	Immunomodulatory activity that might be related to interactions with monocytes-macrophages and T-helper cells, especially Th1-like cells *in vitro*	[65]
*β*-Casein	f54–59	Stimulates phagocytosis of SRBCs by murine macrophages *in vitro*; significantly enhance the resistance of mice to normally lethal infection with *K. pneumoniae*	[42]
*β*-Casein	f54-59 (Gly-Leu-Phe)	Stimulates phagocytosis of SRBCs and provides protection against infection by *Klebsiella pneumonia in vivo*	[73]
*β*-Casein	f191–193 (Leu-Leu-Tyr)	Fails to protect mice against infection but slightly but significantly stimulates antibody secretion against SRBCs by murine spleen cells *in vivo*	[73]
*β*-Casein	FLAb	Has immunomodulatory activity that might be related to interactions with monocytes-macrophages and T-helper cells, especially Th1-like cells	[74]
*β*-Casein	f193-209	Upregulates MHC class II antigen expression on bone marrow-derived macrophages, increasing their phagocytic activity, and induces only a low level of cytokine release	[75]
*β*-Casein	HLGG	Suppresses the proliferation of lymphocytes	[65]
*β*-Casein	Pancreatin/trypsin-derived peptides	Inhibits mitogen-stimulated proliferative responses of murine splenic lymphocytes and rabbit Peyer's patch cells when included in cell culture *in vitro*	[48]

*κ*-Casein∗, bovine *κ*-casein; HLGG, hydrolysed by *Lactobacillus GG*; dPHLr, derived peptides by hydrolysis with *Lactobacillus rhamnosus*; LPS, lipopolysaccharide; PHA, phytohaemagglutinin; SRBCs, sheep red blood cells; FLAb, fermented by lactic acid bacteria.

**Table 4:** Types of TLRs and OPRs in leukaemia cells.

Cell type	Opioid receptor	TLR receptor	Ref
Jurkat leukaemia cell line	MOR	—	[123]
Acute lymphoblastic leukaemia	MOR	—	[124]
HL60 leukaemia cell line, T-cell lymphoblastic leukaemia cells	MOR	—	[125]
AML M4 and M5	—	TLR4	[126]
Jurkat, K562 and HL-60 leukaemia cell lines	—	TLR4	[127]
AML M3	—	TLR4 y TLR2	[128]
THP-1 and HL-60 leukaemia cell lines	—	TLR4	[129, 130]

AML, acute myeloid leukaemia; MOR, *µ*-opioid receptor; TLR, toll-like receptors.
